# Supplementary material for: Analysis of PPARγ Signaling Activity in Psoriasis
Source: Int J Mol Sci. 2021 Aug 10;22(16):8603. doi: 10.3390/ijms22168603 (PMC8395241; doi:10.3390/ijms22168603)
Supplement: Supplementary file 1 [file ijms-22-08603-s001.zip › Supplemental materials_Analysis of PPARg signaling activity in psoriasis/Pathway models/Models images and html files/PPARG inhibits phenotypes assotiated with psoriasis/132847.html]

vascular disease


# Disease vascular disease

|  |  |
| --- | --- |
| URN | urn:agi-meshdis:Vascular%20Diseases |
| Total Entities | 51 |
| Connectivity | 4829 |
| Name | vascular disease |

---

|  |  |
| --- | --- |
| ChildConcepts | thrombosis and embolism |
|  | coronary artery disease |
|  | spinal cord vascular disease |
|  | cerebrovascular disease |
|  | peripheral vascular disease |
|  | arterial disease |
|  | aneurysm |
|  | vascular neoplasm |
|  | vasoplegia |
|  | vessel perforation |
|  | vascular injury |
|  | vessel rupture |
|  | venous disease |
|  | endoleak |
|  | endothelial lesion |
|  | extravasation |
|  | pulmonary vascular disease |
|  | spontaneous portosystemic shunt |
|  | vascular fibrosis |
|  | vascular necrosis |
|  | vasomotor disorder |
|  | intimal hyperplasia |
|  | portal hypertension |
|  | vascular access failure |
|  | blood vessel degeneration |
|  | vasospasm |
|  | diabetic angiopathy |
|  | graft occlusion |
|  | saphenous vein graft disease |
|  | intravascular papillary endothelial hyperplasia |
|  | vascular calcification |
|  | rare circulatory system disease |
|  | vascular lesion |
|  | vascular dysfunction |
|  | blood vessel ingrowth |
|  | vascular infiltration |
|  | vascular invasion |
|  | capillary fragility |
|  | vascular remodeling |
|  | vascular distension |
|  | vascular infarction |
|  | vascular stenosis |
|  | vascular hyperpermeability |
|  | vascular thickening |
|  | endothelial cell dysfunction |
|  | arteriolopathy |
|  | arteriovenous nicking |
|  | leukoariosis |
|  | vascular complication |
|  | vascular stiffness |
|  | vasomotor symptoms |

---

|  |  |
| --- | --- |
| Pathway | PPARG inhibits phenotypes assotiated with psoriasis |
|  | efects selected |
|  | Neighbors of vascular disease |

---

|  |  |
| --- | --- |
| MedScan ID | 9000177 |

---

|  |  |
| --- | --- |
| Alias | vascular disturbance |
|  | vascular problems |
|  | vascular pathogeneses |
|  | vascular disturbances |
|  | vascular derangements |
|  | disorder of blood vessels |
|  | angiopathy |
|  | macroangiopathies |
|  | disorders of blood vessels |
|  | vasculopathy |
|  | vascular deterioration |
|  | disorder of blood vessel |
|  | hemangiopathies |
|  | vascular syndrome |
|  | macroangiopathy |
|  | pathogeneses of the vascular |
|  | blood vessel diseases |
|  | pathogenesis of the vascular |
|  | vascular pathology |
|  | vasculopathia |
|  | vascular disease |
|  | ischemic vasculopathiesy |
|  | vascular disorders |
|  | vascular pathogenesis |
|  | vascular pathologies |
|  | vascular problem |
|  | vascular events |
|  | ischemic vasculopathy |
|  | blood vessel disorders |
|  | blood vessel disease |
|  | blood vessel disorder |
|  | vascular disorder |
|  | vascular deteriorations |
|  | ischemic vasculopathies |
|  | vasculopathies |
|  | macro-angiopathy |
|  | angiopathies |
|  | vascular event |
|  | hemangiopathy |
|  | vascular syndromes |
|  | vascular diseases |
|  | disorders of blood vessel |

---

|  |  |
| --- | --- |
| MeSH ID | D014652 |
|  | M0022535 |

---

|  |  |
| --- | --- |
| MeSH Heading | Vascular Diseases |

---
